# Using Satellite and Airborne LiDAR to Model Woodpecker Habitat Occupancy at the Landscape Scale

**DOI:** 10.1371/journal.pone.0080988

**Published:** 2013-12-06

**Authors:** Lee A. Vierling, Kerri T. Vierling, Patrick Adam, Andrew T. Hudak

**Affiliations:** 1 Department of Forest, Rangeland, and Fire Sciences, McCall Outdoor Science School, University of Idaho, Moscow, Idaho, United States of America; 2 Department of Fish and Wildlife Sciences, University of Idaho, Moscow, Idaho, United States of America; 3 Environmental Science, University of Idaho, Moscow, Idaho, United States of America; 4 Rocky Mountain Research Station, US Forest Service, Moscow, Idaho, United States of America; University of California, Berkeley, United States of America

## Abstract

Incorporating vertical vegetation structure into models of animal distributions can improve understanding of the patterns and processes governing habitat selection. LiDAR can provide such structural information, but these data are typically collected via aircraft and thus are limited in spatial extent. Our objective was to explore the utility of satellite-based LiDAR data from the Geoscience Laser Altimeter System (GLAS) relative to airborne-based LiDAR to model the north Idaho breeding distribution of a forest-dependent ecosystem engineer, the Red-naped sapsucker (*Sphyrapicus nuchalis*). GLAS data occurred within ca. 64 m diameter ellipses spaced a minimum of 172 m apart, and all occupancy analyses were confined to this grain scale. Using a hierarchical approach, we modeled Red-naped sapsucker occupancy as a function of LiDAR metrics derived from both platforms. Occupancy models based on satellite data were weak, possibly because the data within the GLAS ellipse did not fully represent habitat characteristics important for this species. The most important structural variables influencing Red-naped Sapsucker breeding site selection based on airborne LiDAR data included foliage height diversity, the distance between major strata in the canopy vertical profile, and the vegetation density near the ground. These characteristics are consistent with the diversity of foraging activities exhibited by this species. To our knowledge, this study represents the first to examine the utility of satellite-based LiDAR to model animal distributions. The large area of each GLAS ellipse and the non-contiguous nature of GLAS data may pose significant challenges for wildlife distribution modeling; nevertheless these data can provide useful information on ecosystem vertical structure, particularly in areas of gentle terrain. Additional work is thus warranted to utilize LiDAR datasets collected from both airborne and past and future satellite platforms (e.g. GLAS, and the planned IceSAT2 mission) with the goal of improving wildlife modeling for more locations across the globe.

## Introduction

Remote sensing data have become fundamental for mapping actual or potential species distributions [1], [2] to aid conservation efforts at local to global scales [3], [4], [5]. Current species distribution maps draw upon a variety of information sources, including land cover, to model habitat. However, land cover classified as forest is often too broadly defined and often does not account for structural variability that can significantly alter habitat suitability for avian species [6]. While passive spectral remote sensing data products are essential for delineating horizontal habitat heterogeneity (e.g. forest fragmentation) and vegetation productivity at spatial scales relevant to many species, they are of limited use for quantifying details of vertical habitat structure crucial to animal habitat (e.g. [7]). As a result, improving maps of animal distributions is likely to require remote quantification of vertical habitat structure across wide areas [7]–[9].

Light detection and ranging (LiDAR) provides unique data for characterizing vertical habitat structure, and has been utilized widely to address multiple animal-habitat relationships. For instance, airborne LiDAR has been used alone or in combination with passive remote sensing data to assist habitat modeling for terrestrial birds [10]–[17], mammals [18], [19], and invertebrates [20], [21] at spatial scales relevant to numerous organisms. The basis for these advances in habitat modeling stem from the fact that the energy returned in the LiDAR signal provides information to characterize forest structural characteristics such as vegetation height, density, and volume in discrete vertical slices above ground level [22], [23]. The resultant data can provide information ranging from structural characteristics of individual trees [24] to area-wide maps of forest successional status [25], [26] and biomass distribution [27], [28]. As a result, these vertical forest characteristics afforded from airborne LiDAR can improve our understanding and mapping of animal-habitat relationships from the stand to the landscape scale [29].

Although LiDAR has provided significant leaps forward in modeling habitat, data are typically collected via aircraft, thereby imposing limits on the extent and frequency of spatial and temporal sampling relative to orbiting sensors mounted on satellites. A satellite based LiDAR remote sensing platform may provide information about 3-D structure for animal habitat modeling critical for broad-scale application [30]. However to our knowledge no empirical study has yet tested the utility of satellite LiDAR for habitat modeling.

The Geoscience Laser Altimeter System (GLAS) LiDAR instrument aboard the ICESat satellite, in operation between 2003 and 2009, provides a novel opportunity for delineating habitat. While the primary intent of the GLAS instrument was to measure polar ice-sheet thickness [31], GLAS data has been used to characterize vertical vegetation structure [32]–[34] and forest biomass [35]. Similar to airborne LiDAR in the underlying physics, a photo-detector within the GLAS receiving telescope records the time-of-flight (*t*) between emission of the transmit laser pulse and its return to the instrument. Using the speed of light (*c*), distance (*D*) from the instrument is then computed as *D* = 0.5 *tc*. Vertical vegetation structure is derived from the varying intensity at discrete heights of the returned transmit signal [34]. The major attraction of GLAS data for informing ecological questions is that (1) the data samples forest environments across the globe and (2) the data (along with computer code to manipulate it) is freely available from the U.S. National Snow and Ice Data Center (NSIDC). However, LiDAR data from GLAS differs in both spatial extent and resolution from airborne LiDAR data. While airborne LiDAR data are typically scanned at high horizontal resolution (i.e. with each laser pulse sampling a ‘small footprint’ <1 m^−2^ in size and typically collected at a density of >1 pulse m^−2^) continuously across landscapes, GLAS is a ‘large footprint’ instrument collecting data samples with a diameter in our study area of ∼64 m each and separated by a minimum of 172 m. The utility of GLAS LiDAR to describe wildlife habitat at spatial scales relevant to animals is as yet unexplored.

Advances in the global description of vertical vegetation structure are increasingly important for understanding issues associated with species distribution, diversity, and habitat connectivity (e.g. [9]). The major objective of this study was to model breeding site occupancy of a forest dependent bird species, the Red-naped Sapsucker (*Sphyrapicus nuchalis*), using both GLAS and airborne LiDAR data. We chose the Red-naped Sapsucker because of its status as an ecosystem engineer, capable of creating cavities in trees that can be used by multiple other forest avian and mammalian species (e.g. [36], [37]). To our knowledge, this study represents the first to utilize spaceborne LiDAR data in assessing the habitat associations of a forest dependent species, and the first to compare GLAS and airborne-LiDAR models in this ecological context.

## Methods

### Study Site

**Figure 1:**
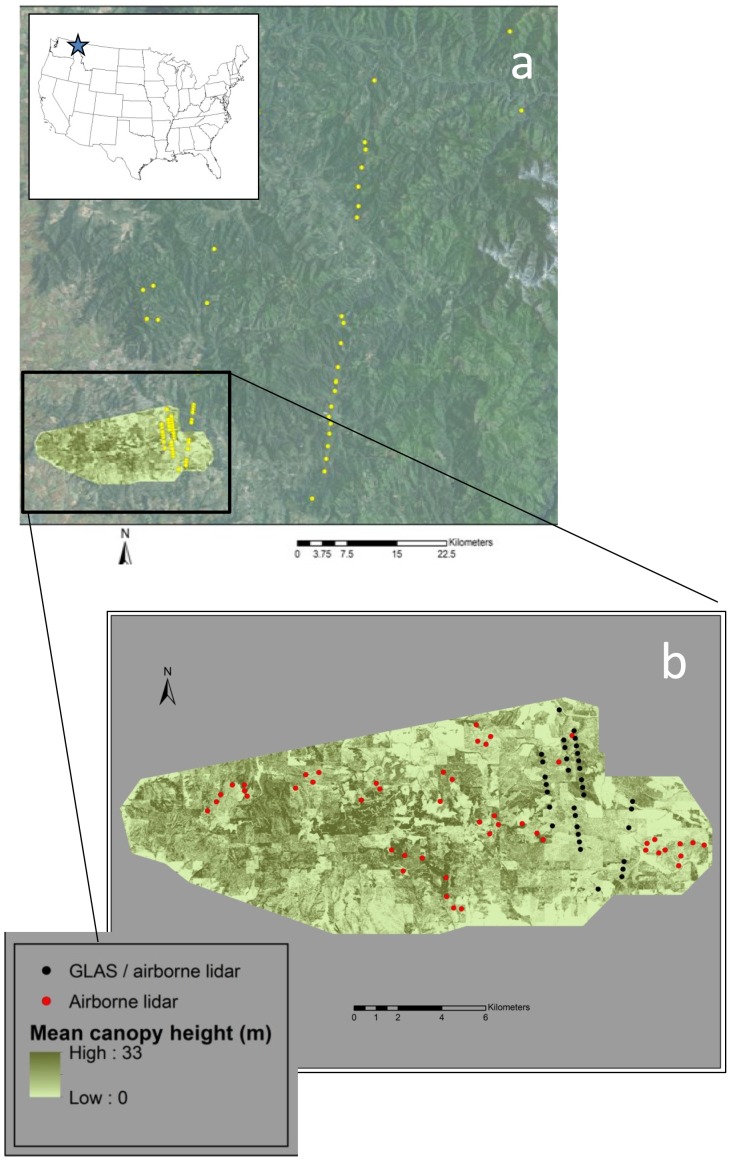
Map of woodpecker survey sites within GLAS LiDAR footprints (yellow dots) (a) in northern Idaho, and (b) survey sites within both GLAS and airborne LiDAR (black dots) and airborne only (red dots).

Field survey sites were located in temperate mixed-conifer forests dominated by Ponderosa pine (*Pinus ponderosa*), White pine (*Pinus monticola*), Grand fir (*Abies grandis*), Western larch (*Larix occidentalis*), Douglas fir (*Pseudotsuga menziesii*), and Western red cedar (*Thuja plicata*) located at the western extent of the Clearwater Mountain Range and within the Idaho Panhandle National Forest in northern Idaho, USA (Fig. 1). Site ground elevation (hereafter, elevation) ranged from 794–1357 m. Land ownership and management across the study area included private timber concessions, private land holders, and state and federal management agencies. Permits are not explicitly required for observational bird studies on U.S. Forest Service land. We obtained verbal permission and access to state owned property from the Idaho Department of Lands. Private land holdings included Potlatch Corporation and Bennett Lumber Products, both of whom granted permission to perform research on their respective properties. The diverse ownership and management matrix has created a mosaic of forest stands that exhibit a range of successional stages that can influence the habitat selection behavior of woodpeckers [38]–[41].

### GLAS LiDAR

The GLAS instrument is a waveform LiDAR system with 15 cm vertical resolution that uses a near infrared (1064 nm) laser. The laser records data covering a ground footprint (i.e. sample area) of ca. 64 m diameter that can vary significantly in its ellipticity over time [42]. The semimajor axis and eccentricity are recorded for each laser shot and were used to define an elliptical mask for extraction of terrain features from the airborne and GLAS data using geographic information system software. The laser footprints are spaced 172 m between centers along the satellite transect, with a between-transect spacing of about 540 m with different acquisition years in our study area (Fig. 1). We acquired GLAS data from the NSIDC, which distributes data products from 18 laser sampling campaigns. We used the GLA01 product (L1A Global Altimetry) which provides the transmitted and received waveform, and the GLA14 product (L2 Land Surface Altimetry) which provides the laser footprint geolocation and geodetic, instrument, and atmospheric corrections. The two datasets are linked by record ID and shot number. We used ‘release 531’ data, which specifies a level of data pre-processing, from laser campaigns 2A (Sep–Nov 2003), 2B (Feb–Mar 2004), 3A (Oct–Nov 2004), 3B (Feb–Mar 2005), 3D (Oct–Nov 2005), 3H (Mar–Apr 2007), and 3I (Oct–Nov 2007). GLAS waveforms can be influenced by interaction with clouds and signals that are saturated due to high gain settings. We therefore removed waveforms that were affected by clouds and saturation on the laser signal following Chen [43].

**Figure 2:**
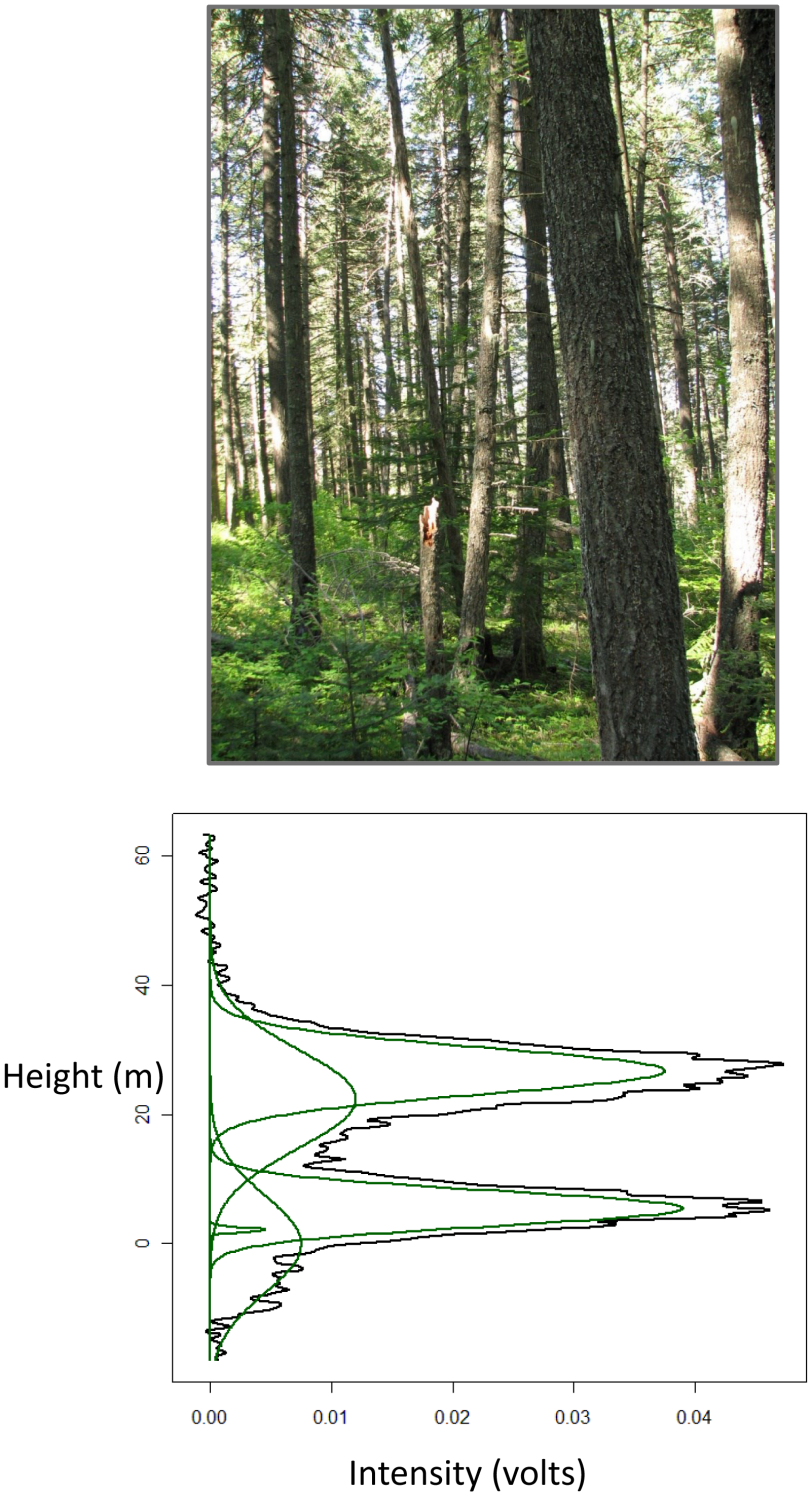
Example GLAS LiDAR waveform signal (bottom panel) and associated woodpecker survey location (top panel). The black line in the graphic represents the actual laser energy signal for the plot location, while the green lines represent the decomposed signal when expressed in best-fit Gaussian curves.

We modified Interactive Data Language (IDL) code provided by NASA and available on the NSIDC GLAS site [44] to extract complete laser waveform return signals (from GLA01) and waveform summary statistics (from GLA14) (Fig. 2). To compute vertical vegetation statistics from the waveform signal, we first established the ground return provided by the summary statistics in the GLA14 product. The summary statistics include up to 6 primary energy peaks generated from a Gaussian decomposition algorithm [45] to identify the major components of the signal which include both vegetation and ground returns (Fig. 2). We estimated the ground return location following Chen [46] who investigated the correlation between GLAS and airborne lidar data in forested mountainous areas and found that the largest in magnitude of the two lowest peaks corresponded most closely with ground elevations.

**Figure 3:**
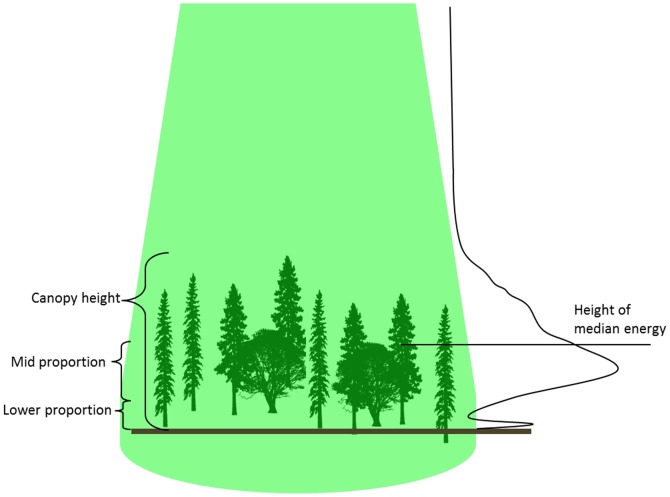
Cartoon depicting locations of vegetation metrics within canopy. The green cone in the figure represents the GLAS laser footprint scale, which subtended an area of roughly 64m diameter. The black curve on the right of the figure represents a stylized GLAS energy return profile for this particular cartoon arrangement of vegetation and ground.

Before deriving canopy height statistics (Fig. 3), we filtered the waveforms by retaining signals that were greater than 4 times the standard deviation of the noise (following [47]). Maximum waveform vertical resolution at 1 ns pulse duration is 15 cm. We selected a Gaussian filter of 60 cm (4 ns) in height because we were interested in primary structural features of the canopy. We did not evaluate other filter widths, but narrower widths may allow noise to pass or may produce results that are difficult to interpret due to their vertical length scale being finer than individual branches. Canopy height was estimated from an algorithm developed by Chen [43] for a coniferous montane location in the Pacific Northwest that accounts for the effect of terrain slope on waveform vegetation indices by incorporating the maximum terrain relief distance within the laser footprint (obtained from the 10 m National Elevation Dataset [48]) and the difference between the start and end of the filtered and smoothed waveform signal. The complete suite of canopy metrics derived from GLAS data is listed in Table 1.

**Table 1:** GLAS LiDAR metrics considered for occupancy analysis; only the metrics denoted by double asterisks (**) were used after collinearity analysis.

Metric abbreviation	Metric
**Elev	elevation (m)*
**HLI	heat load index*
**p.lower	proportion of total return energy that represents vegetation between 0 and 3 m above ground level (agl)
**p.mid	proportion of total return energy that represents vegetation between 3 and 10 m agl
**can.height	canopy height computed from Chen (2010b) algorithm
ht.mean	mean canopy height agl
ht.var	variance of vegetation heights
ht.cv	coefficient of variation of vegetation heights
ht.med	median canopy height
ht.mad	median absolute deviation from median of canopy heights
Vdr	vertical distribution ratio (can.height – HOME)/can.height
**veg.density	canopy density – proportion of return energy representing vegetation to total return energy that includes ground returns
**HOME	height of median energy density
**FHD	foliage height diversity (1 m bin); diversity in vegetation distribution. See MacArthur & MacArthur, 1961

These metrics include 6 biotic factors (p.lower, p.mid, can.height, veg.density, HOME, and foliage height diversity (FHD)) and 2 abiotic factors (heat load index (HLI) and elevation.

* Heat load index integrates measures of site slope, aspect, and latitude, computed following [81]. Note that due to difficulties in deriving slope using GLAS data, HLI and elevation were derived from the 10-m National Elevation Database.

### Airborne LiDAR

LiDAR data for Moscow Mountain (Fig. 1) was acquired in July, 2009 using a discrete return airborne Leica ALS50 Phase II laser system scanning with a pulse rate of ∼150 kHz at ±14° from nadir. Adjacent survey transects were flown in opposing directions with ≥50% overlap to ensure 100% terrain overlap for all transects, resulting in a mean data density of ∼12 points m^−2^. A maximum of 4 range measurements, or returns, were recorded for each laser pulse transmission. To geolocate laser point positions, aircraft attitude (pitch, roll, yaw) was recorded at 200 Hz by an onboard inertial measurement unit (IMU), while sensor position (x, y, z) was recorded at 2 Hz by an onboard differential GPS. Aircraft attitude and sensor position were indexed by GPS time to enable post-processing correction and calibration. Data were projected in UTM Zone 11 North, NAD 83 using NAVD88 Geoid 83 for the vertical datum and NAD83 for the horizontal datum.

The Multiscale Curvature Classification algorithm [49] was used to delineate ground from non-ground coordinate corrected LiDAR data. Returns classified as ground were interpolated into a 1 m resolution DEM and subtracted from non-ground classified points to generate a topographically normalized point cloud. Metrics describing vegetation structural characteristics were derived from all points greater than 1 m in height within 20 m grid cells (see Table 2 for a list of all metrics derived by airborne LiDAR data). The 20 m grid cell resolution was used so that the airborne LiDAR metrics could be compared with and validated against measurements of forest structure collected in the field (see [28]). The resultant 20 m raster grids, each representing a vegetation height metric, were downsampled to 1 m resolution in the vicinity of the GLAS LiDAR footprints to minimize the effect of sampling incomplete pixels around the perimeter of the GLAS footprint. Grid cells within a 65 m diameter circle centered on the woodpecker survey site were aggregated to represent the mean value of each vegetation metric and to coincide with the GLAS footprint.

**Table 2:** Airborne LiDAR metrics considered for occupancy analysis; only metrics denoted by double asterisks (**) were used after collinearity analysis.

Metric abbreviation	Metric
**Elev	elevation (m)*
**HLI	heat load index*
p.lower	proportion of total return energy that represents vegetation between 0 and 3 m above ground level (agl)
**p.mid	proportion of total return energy that represents vegetation between 3 and 10 m agl
p.upper	proportion of total return energy that represents vegetation between 10 and 30 m + agl
VDR	Vertical distribution ratio (Goetz *et al.* 2007)
Density	canopy density – proportion of vegetation to total energy return
Hmax	maximum canopy height
Hmedian	median canopy height
Hsd	standard deviation of heights
Hmean	mean canopy height
Crr	canopy Relief Ratio (HMEAN – HMIN)/(HMAX – HMIN)
Hirq	inter-quartile range of heights
**Hmode	height of dominant mode (vegetation density) within return signal
**Hmrange	range between minimum and maximum modes
**Hskew	skewness of height profile across vegetation returns
Hkurt	kurtosis of height profile across vegetation returns
**Hvar	variance of vegetation density weighted height values
Hmad	median absolute deviation from median height
**FHD	foliage height diversity (1 m bin) see MacArthur & MacArthur, 1961

* Heat load index integrates measures of site slope, aspect, and latitude, computed following [81]. Note that while these measures are attainable using airborne lidar, to be consistent with calculations made for GLAS data HLI and elevation were derived from the 10-m National Elevation Database.

### Woodpecker Surveys

We chose Red-naped Sapsuckers as the focus of our study for multiple reasons. As part of a cavity-excavating guild, sapsuckers are an ecologically important group owing to their status as ecosystem engineers [36]. Sapsuckers and other woodpeckers create keystone structures (i.e. cavities) that many other animals use for nesting and/or roosting [37], [50]–[52]. In addition, cavity excavators in general rely upon a set of forest structural characteristics that may be described via LiDAR. For instance, Red-naped Sapsuckers forage in areas with a sparse understory, and in sections of the canopy that are relatively open to facilitate insect gleaning on trunks [53]. Additionally, the diameter of the nest tree, the fraction of canopy cover, and the distribution of vegetation are all important nest site attributes [54] and these are all potentially quantifiable metrics from both airborne and spaceborne LiDAR data.

Eighty two woodpecker survey sites were subsampled randomly from within the airborne LiDAR flight during 27 May–3 July 2009 from existing song bird point count locations (Fig. 1) and within seventy three GLAS spaceborne LiDAR footprints during 19 May–2 July 2010. A subset of survey sites (n = 39) occurred within both spaceborne and airborne LiDAR data samples (Fig. 1). To ensure that we were not double-counting the same birds, we selected sites with a minimum of 340 m spacing (equating to ∼12 ha surrounding each survey location) which reflects the mean observed home range of Red-naped Sapsucker [53]–[55]. Surveys conducted outside of GLAS LiDAR sites were conducted to increase sample size for the airborne LiDAR derived occupancy model.

Woodpecker surveys were conducted following Drever *et al.* [56] using playback recordings to elicit responses from woodpeckers. Two identical playbacks, each comprised of a call and drum, were played for Red-naped Sapsuckers, with a two minute silence in between, and all Red-naped Sapsuckers seen or heard were recorded. Because the purpose of the surveys was to establish breeding bird-habitat relationships, we constrained the dates of the surveys to correspond to the breeding season of this species [55], [56]. This timing is important because the use of callbacks is only effective while the birds are breeding and territorial. Each survey point was visited twice during the breeding season because multiple visits are necessary in order to better evaluate detection issues (i.e. when birds are really present but are recorded as absent [57]). Issues of detectability were explicitly incorporated into our analysis (see below). Surveys conducted solely within GLAS footprints were completed in the Idaho Panhandle National Forest (Fig. 1) in 2011 from 19 May–27 June. We selected survey sites by comparing the 2009 airborne LiDAR dataset to an earlier airborne LiDAR dataset flown in 2003 (see [28] for complete description of this multi-temporal analysis) to ensure that harvest operations had not taken place spanning the period from the first GLAS data collection to the date of the airborne LiDAR data acquisition.

### Data Analysis

We used a t-test to compare the vegetation metrics between occupied and unoccupied sites, and considered p≤0.05 to be statistically significant. Our model development was conducted in an exploratory, rather than confirmatory, framework. We utilized this exploratory approach because Walters *et al.* [58] notes that breeding habitat relationships for Red-naped Sapsucker have been conducted where aspen (*Populus tremuloides*) and deciduous forests are important components of the forest, and our study sites contained very little deciduous forest. Preliminary data analysis revealed multi-collinearity among several GLAS and airborne LiDAR derived vegetation height and density metrics (Tables 1 and 2). We computed condition indices and variance decomposition proportions following Belsley [59] to identify the information redundant covariates. After applying conditioning diagnostics, our final set of site covariates included 6 biotic and 2 abiotic variables for the GLAS LiDAR based model (Table 1) and the airborne LiDAR based model (Table 2). We included elevation and heat load index to account for abiotic factors that may influence nest or foraging site selection. The 6 biotic variables are represented by the LiDAR-derived forest structure metrics, while elevation and heat load index were derived from the 10 m National Elevation Dataset [37].

While species presence and absence may be modeled with logistic regression, the approach assumes equal probability of detection across sites and cannot account for detection probabilities less than 1 [57], [60], [61]. Imperfect detection (i.e. *p*<1) produces biased estimates of latent variables that describe the state processes typically of interest [62], [63]. We used a hierarchical occupancy model that explicitly accounts for detection probability that is conditional on the state process controlling species occurrence as implemented in the R package [64] ‘unmarked’ [65]. Due to the relatively small number of sample sizes for presence/absence modeling for each LiDAR dataset (airborne: n = 82; GLAS: n = 73), we pooled responses from the Moscow Mountain and the Idaho Panhandle National Forest regions for use in a single season occupancy model. Variables from Table 1 were used to model the latent occupancy state of each site. To account for detection probability, we included Julian day and a categorical measure of weather conditions (e.g. wind, cloud cover) where increasing values were associated with an increase in those weather variables.

We computed global, null, and reduced occupancy models for Red-naped Sapsuckers and evaluated relative model performance using Akaike’s Information Criterion corrected for small sample size (AICc) [66]. We allowed for all linear combinations of predictor variables, and excluded models where the only predictor variables were the detectability covariates, requiring at least one process variable (vegetation, heat load index, or elevation) to be present. This method generated a list of models that struck a balance between including all possible habitat structure combinations, while excluding ecologically irrelevant models [67].

Global (full) model fit and checks for overdispersion were evaluated using a likelihood ratio test procedure to compute the deviance between the saturated model and the full model. The deviance was then divided by the residual degrees of freedom (*n*–*p* = 72) to assess fit and potential overdispersion. We performed all first-order subsets regression to determine the top candidate models; while many studies utilize ΔAICc<2, we opted to utilize a more conservative ΔAICc<6 for final model selection evaluation [68], [69]. Where model selection uncertainty was high (e.g. weights were <0.3) [66], we utilized a model-averaging approach. Parameter estimates in model averaging represent the sum of the product of the parameter estimates in each model with their associated model weight. As model weight tends towards zero, so too does the value of the parameter estimate. The model-averaging approach generates a new set of parameters that are used to replace the original parameter estimates in the global model. The procedure is recommended when all-subsets modeling is performed [70] and when strong support is lacking for a single model [66], [71].

Relative variable importance was computed from model averaged parameter estimate weights to ascertain the contribution of each variable to the averaged model [66]. We believe our approach to model development addresses the concerns raised by Murtaugh [72] and Dahlgren [73], specifically with regards to shrinkage of estimates derived from model averaging that contribute the least amount of information to the model.

### Model Cross Validation and Predictive Power

We assessed model performance using the Area Under the Curve (AUC) from the Receiver Operator Characteristic (ROC) curve where presence/absence probability cut-off values were determined using *k*-means cluster analysis by partitioning the predicted probabilities into 2 groups. We generated AUC scores first with unvalidated data, and then generated AUC scores using leave-one-out cross validation (LOOCV) to assess the degree of over-fitting and model sensitivity. AUC values close to 0.5 represent a model whose predictions of presence/absence are no different than random.

## Results

We detected Red-naped Sapsucker responses at 48% (39 out of 82) of sites surveyed within area covered by airborne LiDAR and at 28% (28 out of 73) of sites surveyed within area covered by spaceborne LiDAR. Of the 155 total survey sites, 39 were common to both airborne and spaceborne LiDAR surveys. Fifteen of 39 Red-naped Sapsucker responses were reported at the coincident airborne and spaceborne LiDAR sites (44%). There were no statistical differences between the occupied and unoccupied sites (Table 3).

**Table 3:** Means and standard errors of GLAS and airborne LiDAR variables used in sapsucker models.

Metrics	Occupied sites (n = 28)		Unoccupied sites (n = 45)	
GLAS (spaceborne LiDAR; n = 73 )	Mean	SE	Mean	SE
Elevation (m)	950	21.1	974	17.8
HLI (heat load index)	0.842	0.014	0.871	0.009
p. lower	0.226	0.025	0.205	0.018
p.mid	0.347	0.026	0.389	0.021
Can.height (m)	34.1	2.06	32.2	1.41
Veg.density	0.897	0.014	0.905	0.011
HOME	10.87	1.02	10.96	0.669
FHD	2.092	0.015	2.11	0.012
	**Occupied sites (n = 39)**		**Unoccupied sites (n = 43)**	
**Airborne LiDAR (n = 82)**	**Mean**	**SE**	**Mean**	**SE**
Elevation (m)	956	13.9	941	12.8
HLI (heat load index)	0.875	0.008	0.845	0.014
p.mid	0.205	0.019	0.239	0.022
Hmode	2.59	0.736	3.80	0.965
Hmrange	18.4	1.00	16.5	1.00
Hskew	1.51	0.244	1.35	0.340
Hvar	56.9	6.33	47.6	5.09
FHD	1.33	0.014	1.34	0.038

### Model Fit and Parameter Estimation

#### GLAS LiDAR

Global model fit was good (D = 82.8) when compared with the residual degrees of freedom (*rdof* = 63) as indicated by the over-dispersion parameter, ĉ (ĉ = 1.3). Because ĉ≈1, there is no reason to doubt the fit of the global model, nor does over-dispersion appear to be large enough to warrant using a quasi AICc framework [65]. Due to high model selection uncertainty as indicated by AICc weights<0.3 (Table 4), we proceeded with a full model averaging approach for models with ΔAIC≤6. Neither the original global model nor the averaged model produced parameter estimates with acceptable confidence as the confidence intervals all included zero (Table 5).

**Table 4:** Occupancy models for Red-naped Sapsuckers with a ΔAICc≤6 based on GLAS LiDAR.

Model #	Elev (m)	FHD	HOME	Canopy height	HLI	p.lower	p.mid	Vegetationdensity	JulianDay	sky	df	logLik	AICc	delta	weight
160	−67.05	37.45	−98.89	203.13	−48.58			31.84			8	−66.60	151.45	0.00	0.177
224	−93.36	61.76	−157.13	285.28	−63.16		−9.75	57.39			9	−65.85	152.56	1.11	0.101
382	−68.41		−153.43	156.54	−77.74	−100.44	−2.72		−0.33		9	−65.97	152.79	1.34	0.090
928	−78.24	43.22	−114.44	236.73	−56.85			36.39	−0.36	−0.19	10	−64.69	152.92	1.47	0.085
672	−77.98	43.53	−115.08	236.15	−56.31			37.07		−0.19	9	−66.16	153.17	1.72	0.075
221			−144.38	174.87	−66.23		−78.37	76.93			7	−68.88	153.47	2.02	0.064
176	−147.04	107.85	−110.06	294.86		69.83		62.13			8	−68.05	154.35	2.90	0.041
894	−106.34		−221.14	247.94	−119.19	−147.19	10.61		−0.32	−0.20	10	−65.49	154.54	3.08	0.038
206	−68.28		−135.07	132.80			−49.41	79.31			7	−69.64	155.01	3.55	0.030
477			−91.61	127.66	−41.94		−45.09	41.18	−0.29		8	−68.44	155.14	3.68	0.028
733			−177.87	216.25	−82.75		−96.36	94.85		−0.18	8	−68.46	155.16	3.71	0.028
192	−62.66	−15.09	−137.55	140.90	−82.43	−106.11		−6.70			9	−67.31	155.47	4.02	0.024
448	−68.42	12.38	−135.16	178.96	−69.03	−64.18		10.00	−0.33		10	−65.97	155.50	4.04	0.023
736	−87.09	70.01	−209.57	296.30	−60.45		−46.08	78.18		−0.21	10	−66.02	155.59	4.14	0.022
223		−33.28	−140.63	197.60	−104.06		−78.12	63.89			8	−68.78	155.81	4.36	0.020
462	−51.77		−103.44	101.11			−38.33	60.88	−0.28		8	−68.79	155.83	4.38	0.020
992	−189.14	121.41	−364.00	606.65	−138.12		−55.46	137.20	−0.32	−0.22	11	−64.77	155.87	4.42	0.019
989			−129.90	157.61	−59.85		−70.45	69.10	−0.29	−0.19	9	−67.55	155.96	4.51	0.019
512	−89.08	41.11	−166.08	248.49	−72.57	−44.97	−1.72	37.27	−0.37		11	−64.95	156.23	4.78	0.016
479		−16.27	−132.75	175.36	−74.68		−77.53	63.72	−0.29		9	−67.75	156.36	4.90	0.015
16	−58.55	40.27	−52.28	88.17							6	−71.58	156.44	4.98	0.015
640	−76.42	2.10	−166.48	180.24	−84.73	−102.70	−3.51			−0.21	10	−66.75	157.04	5.59	0.011
704	−56.05	0.02	−107.61	137.96	−63.41	−64.87		3.77		−0.21	10	−66.79	157.13	5.68	0.010
718	−53.67		−107.68	105.14			−39.86	63.27		−0.15	8	−69.47	157.19	5.74	0.010
768	−62.97	30.52	−102.80	185.63	−49.88	−13.95	−1.48	28.47		−0.19	11	−65.43	157.19	5.74	0.010
238	−50.93		−102.06	107.28		7.77	−41.17	63.69			8	−69.53	157.30	5.85	0.009

**Table 5:** Model averaged parameter estimates, standard errors, and 95% confidence intervals using GLAS LiDAR data.

Parameter		Estimate	Std. Error	Lower CI	Upper CI
Occupancy	elev	69.6	116	−157	296
	FHD	110	291	−461	681
	HLI	56.5	196	−327	440
	hmode	−59.4	87.4	−231	112
	hmrange	82.8	179	−268	434
	hskew	112	316	−507	731
	hvar	−50.7	103	−253	152
	p.mid	19.7	74.5	−126	166
Detection	wind	−0.26	0.20	−0.64	0.13

#### Airborne LiDAR

Global model fit for Red-naped Sapsuckers indicated that the selection and number of parameters were adequate to model the data compared to that of a saturated model. The deviance was 76.6 and the overdispersion parameter (*c-hat*) = 1.05 with 72 residual degrees of freedom (*dof = n−p*). Model selection uncertainty in the airborne LiDAR derived models was high according to AICc model weights so we performed full model averaging including models with AICc≤6 (Tables 6 and 7).

**Table 6:** All subsets models for Red-naped Sapsuckers with ΔAIC≤6 based on airborne LiDAR data.

Model #	Elev	FHD	HLI	hmode	hmrange	hskew	hvar	p.mid	wind	df	logLik	AICc	delta	weight
384	70.50	115.97	27.05	−66.44	82.84	83.16	−54.48		−0.25	10	−79.57	182.24	0.00	0.44
512	78.33	113.98	30.32	−63.20	92.53	92.54	−53.74	17.49	−0.25	11	−79.47	184.70	2.47	0.13
448	73.00	90.95	24.19	−49.88	55.74	111.96		23.54	−0.30	10	−81.13	185.35	3.12	0.09
120	21.96	107.30	65.65		73.64	88.37	−21.12			8	−84.19	186.36	4.12	0.06
188	108.37	193.01		−73.42	140.18	327.12		64.45		8	−84.34	186.66	4.42	0.05
7		−28.76	153.00							4	−89.12	186.76	4.52	0.05
63		339.22	196.97	37.61	145.38	371.68				7	−85.96	187.43	5.20	0.03
263		−34.82	186.06						−0.19	5	−88.53	187.84	5.61	0.03
56	34.05	170.41	111.76		106.84	144.86				7	−86.22	187.96	5.72	0.03
135		−29.03	133.33					5.20		5	−88.60	187.99	5.76	0.02
37			142.39			33.49				4	−89.76	188.04	5.81	0.02
149			95.41		−54.03			−58.84		5	−88.64	188.08	5.84	0.02
124	63.46	76.63		−51.78	63.56	51.64	−34.86			8	−85.08	188.13	5.89	0.02

**Table 7:** Original global model parameter estimates, standard errors, and 95% confidence intervals using airborne LiDAR data.

Parameter		Estimate	Std. Error	Lower CI	Upper CI
Occupancy	Elev*	78.3	248	61.7	112
	HLI*	30.3	91.3	16.9	47.9
	p.mid	17.5	98.0	−17.6	41.2
	FHD*	114	301	92.9	146
	Hmode*	−63.2	166	−80.2	−53.0
	Hmrange*	92.5	343	48.0	138
	Hskew*	92.5	268	61.2	141
	Hvar*	−53.7	212	−79.1	−27.9
Detection	wind	−0.25	0.20	−0.65	0.12

* Confidence intervals do not include zero.

Foliage height diversity (FHD) was highly influential to the averaged model (RVI = 0.95) (Table 8) and positively associated with Red-naped Sapsucker occupancy. Other variables that were important for this species included heat load index, the distance between the lowest and highest vegetation biomass (hmrange), and the distribution of distribution throughout the canopy (hskew). The positive association with hmrange indicated that occupancy increased with a greater vertical distance between the minimum and the maximum nodes of vegetation biomass. Visually, this would be represented by a gap in the vegetation structure at an intermediate height between the biomass mode nearest to the ground (the minimum mode likely representing the shrub layer) and the biomass mode in the canopy (the maximum mode likely representing the canopy). The positive relationship with hskew indicated that there is more vegetation closer to the ground than in the upper canopy. The proportion of vegetation in the mid-story (2.5–10 m) was positively associated with Red-naped Sapsucker presence but contributed the least to the averaged model (RVI = 0.32). Wind was a detectability covariate, and occupancy was negatively associated with high wind.

**Table 8:** Relative variable importance (RVI) from model averaging.

Parameter	RVI
FHD	0.95
HLI	0.93
hmrange	0.88
hskew	0.88
elev	0.82
hmode	0.77
wind	0.69
hvar	0.65
p.mid	0.32
julDay	–
sky	–

### Model Cross Validation and Predictive Power

**Figure 4:**
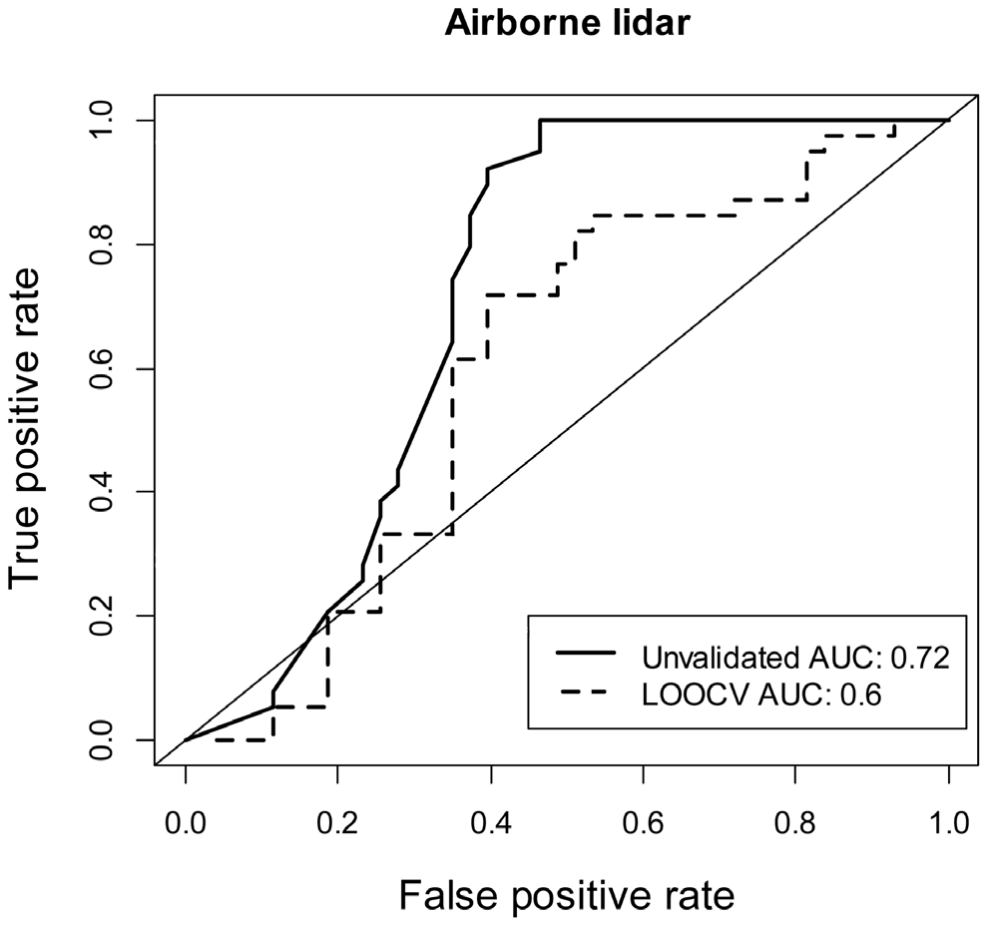
Receiver operator curves with unvalidated and leave-one-out (LOOCV) cross validation for airborne LiDAR.

Due to the high uncertainty associated with our spaceborne models, we did not proceed with an assessment of model performance based on spaceborne LiDAR. The AUC score for the airborne data indicated that the global model performed better than random, with AUC scores ranging between 0.6 and 0.72 (Fig. 4).

## Discussion

This study was novel for two reasons. First, this study addressed whether GLAS spaceborne LiDAR data could be used for wildlife habitat application in the case of one bird species known to be an ecosystem engineer important to creating habitat for other forest species in the Inland Northwest, USA. Secondly, we were able to compare GLAS and airborne LiDAR models developed at the same spatial scale; no studies to date have compared these LiDAR data sets in the context of wildlife habitat assessment. LiDAR data used in this study were averaged over an area of approximately 0.3 ha centered on the survey point. While it is unlikely that the survey points were coincident with the centers of the home ranges of individual birds, the 0.3 ha sampling unit coincided with the size of the GLAS footprint. Thus, we limited our analyses to this spatial scale in order to facilitate comparisons between the two LiDAR data sets as applied to Red-naped Sapsucker habitat.

The model results based upon GLAS spaceborne LiDAR were weak, which may be due to several reasons. First, we had relatively small sample sizes; although we gathered data across three years, fewer birds were detected during 2011. This was a wet summer, and few birds were noted in our expanded GLAS survey sites. Additionally, a scale mismatch may have occurred between the spatial distribution and extent of GLAS footprints and variables that influence Red-naped Sapsucker habitat selection, which is likely to have affected both the GLAS results as well as the performance of the airborne LiDAR models. Indeed, red-naped Sapsucker may respond to habitat features at multiple spatial scales [58], and recently Sadoti and Vierling [74] noted that features at the home range scale (∼9.1 ha) were most important in a multi-scale analysis of selection. Multiple studies have been conducted on Red-naped Sapsucker breeding habitat in aspen forests [74], [75], but features that might influence Red-naped Sapsuckers in conifer-dominated forests and the associated important spatial scales are relatively unknown [76]. Other studies have noted that woodpeckers select habitat features at multiple spatial scales, and these spatial scales range from nest tree characteristics to landscape features [74], [75], [77], [78]. We were constrained by the spatial distribution and extent of GLAS footprints, and were thus unable to explore the effects of multiple scales. Furthermore, factors within the GLAS footprints that were not detectable might have heavily influenced Red-naped Sapsucker occupancy (e.g. the presence of fungal conks; [75]).

The most important structural variables that influenced Red-naped Sapsucker selection based on airborne LiDAR data included foliage height diversity, the distance between major components in the canopy profile, and the amount of vegetation near the ground. When considered jointly, these three metrics describe a vegetation profile that is uniform throughout its height except for a larger contribution of vegetation near the ground. Red-naped Sapsuckers have been observed to inhabit montane conifer forests, and Walters [53] notes that increased foraging preference was observed in areas of the canopy with fewer branches. Conifer stands with an even distribution of foliage along the height of the forest may represent branch structure that is more open and accessible for foraging. Additionally, a larger contribution of vegetation near the ground, likely representing shrub growth and could support insects. The presence of shrub growth likely supports an increased insect population, and Red-naped Sapsuckers will utilize a combination of flycatching and gleaning to forage during the breeding season [58], [74].

The presence of Red-naped Sapsuckers was also positively associated with increasing elevation and heat load index, although their effect sizes were the smallest. Higher heat indices indicate a preference for sites with warmer combinations of ground slope and aspect. Studies have noted tree cavity entrances located on southern aspects in British Columbia [53], and Colorado and Wyoming [79], to no apparent directional preference in Montana [54]. However, while Martinuzzi *et al*. [18] found that slope and aspect could affect the presence of forest snags near the study area, we are not aware of any studies that indicate slope or aspect preference for the nest tree location within the landscape.

Our results demonstrated that small-footprint airborne LiDAR data were able to provide ecologically meaningful information relative to occupancy for Red-naped Sapsuckers when aggregated and analyzed at a scale similar to that sampled by the large-footprint GLAS satellite LiDAR. This finding indicates that the forest structural metrics calculable from airborne LiDAR were likely more suitable for modeling habitat of this bird than GLAS-based structural metrics at this spatial scale. However, because not all samples contained coincident airborne and satellite lidar, this finding requires further testing at additional field sites and wildlife species, and underscores the need for denser spatial sampling in future satellite-based LiDAR missions for application to mapping and modeling wildlife distributions (see [30]). While the large footprint and non-contiguous nature of GLAS data poses significant challenges for wildlife distribution modeling, these data do provide useful information on ecosystem vertical structure, particularly in areas of gentle terrain (e.g. [80]). We therefore encourage the remote sensing and wildlife communities to join efforts so that additional progress can be made to incorporate LiDAR datasets collected from both airborne and past and future satellite platforms (e.g. GLAS, and the planned IceSAT2 mission) with the prospect of improving wildlife biodiversity and single-species modeling for more locations across the globe.
